# The role of soils in habitat creation, maintenance and restoration

**DOI:** 10.1098/rstb.2020.0170

**Published:** 2021-09-27

**Authors:** Gerlinde B. De Deyn, Lammert Kooistra

**Affiliations:** ^1^ Soil Biology Group, Environmental Sciences, Wageningen University, Droevendaalsesteeg 3, 6700PB Wageningen, The Netherlands; ^2^ Laboratory of Geo-Information Science and Remote Sensing, Wageningen University and Research, Wageningen, The Netherlands

**Keywords:** land degradation, plant–soil feedback, remote sensing, soil biodiversity, soil carbon, soil regeneration

## Abstract

Soils are the fundament of terrestrial ecosystems. Across the globe we find different soil types with different properties resulting from the interacting soil forming factors: parent material, climate, topography, organisms and time. Here we present the role of soils in habitat formation and maintenance in natural systems, and reflect on how humans have modified soils from local to global scale. Soils host a tremendous diversity of life forms, most of them microscopic in size. We do not yet know all the functionalities of this diversity at the level of individual taxa or through their interactions. However, we do know that the interactions and feedbacks between soil life, plants and soil chemistry and physics are essential for soil and habitat formation, maintenance and restoration. Moreover, the couplings between soils and major cycles of carbon, nutrients and water are essential for supporting the production of food, feed and fibre, drinking water and greenhouse gas balances. Soils take thousands of years to form, yet are lost very quickly through a multitude of stressors. The current status of our soils globally is worrisome, yet with concerted action we can bend the curve and create win–wins of soil and habitat conservation, regeneration and sustainable development.

This article is part of the theme issue ‘The role of soils in delivering Nature's Contributions to People’.

## 1. Introduction

As humans we typically have an aboveground macroscopic view of the world around us. Based on the landscape we see we distinguish different types of natural habitats and different types of man-made systems. Each of these habitats is characterized by the species composition that makes up the vegetation, and associated with this vegetation the higher trophic levels that thrive in that habitat with its distinct features. At global scale, we can distinguish major habitat types, the so-called biomes, that occur in zones across the globe with specific combinations of temperature and precipitation in which plant species with particular life-history traits that provide adaptation to the climatic conditions cooccur [1]. Soils are an important modifier of the occurrence of different biomes as water availability is not just a function of precipitation, but rather a function of water availability in the soil and this is strongly modified by soil texture, soil structure, soil depth and organic matter content [2]. Moreover, within the same climatic zone other soil characteristics such as soil pH and soil nutrient availability are also important factors in driving plant species composition because these soil parameters select for plants with specific eco-physiological traits [3]. Differences in these soil parameters result from differences in parent material of the bedrock, soil age, climate, relief and the organisms [4].

Clearly, soil characteristics play a very important role in habitat creation, not only as seen aboveground but also for life belowground. For a long time, soil life largely escaped the attention of naturalists and soil scientists as the vast majority of life in the soil is microscopic, cannot be cultured and lives hidden in the opaque soil substrate. Yet soil life is very fascinating and important, as already recognized by Darwin with his work on earthworms [5]. Over the last decades, new techniques to study soil life and especially microscopic life have revolutionized our view on soil diversity. We now know that soils and all life within are not static but highly dynamic at a range of temporal and spatial scales [6,7]. Soil life not only plays an important role in the functioning and maintenance of soils through the feedbacks between soil organisms and soil chemical and physical properties in present soil. Soil life also plays an important role in soil formation, a process that takes centuries to thousands of years and a succession of different interlinked plants and soil biota. Insight in the interlinkages is of importance to understand the impact of human modification of habitats on soil diversity and functioning and to devise strategies to counteract soil loss and promote soil and habitat restoration.

In this manuscript, we aim to present how soils are a habitat for many species and how in turn soil biota play key roles in soil formation and habitat creation. Next we discuss how humans have been modifying habitats and soils since the onset of agriculture and urbanization and reflect on the current status of our soils. In the last section, we focus on soil and habitat conservation and regeneration and present our views on how with concerted action across science, technology, policy, practitioners and citizens we can bend the curve and create win–wins for soil and habitat conservation, regeneration and sustainable development.

## 2. Soils: habitat for many species

Soils are composed of mineral and organic particles that are arranged in a three-dimensional structure composed of particles and between these particles voids that are filled with air and water. This composition enables soil life to live in the voids, a physical space to hide from predators and adverse aboveground conditions, to obtain water, nutrients and oxygen, and to reproduce. In terrestrial systems, life belowground is more diverse than aboveground [8,9]. The vast majority of terrestrial plants are rooted in soil, start their life cycle in soil and have latent offspring resting in soil (e.g. as seeds) until the conditions become favourable to sprout. Not only plants, also many other organisms have life stages in the soil. Typical examples comprise eggs and larval stages of many insect species that find shelter and food in the soil during this vulnerable life episode. Next to organisms living partly in soil, soils are a habitat to a wide range of organisms that spend their whole life in or dwelling on the soil. These organisms range vastly in size from macroscopic vertebrates and invertebrates (e.g. earthworms), to microscopic invertebrates (e.g. nematodes), and fungi and prokaryotes of just a few micrometres [9].

At global scale, soils harbour millions of species; generally the smaller they are the larger their diversity, yet the less we know about their ecology and global distribution [8,9]. It also has to be noted that our global picture is incomplete as large land areas have not yet been sampled especially in certain continents, e.g. Africa and South America [9,10]. To some extent soil biota distribution is controlled by similar environmental factors as plants, notably by climate, soil texture, pH, nutrient levels, soil humidity, salinity and levels of disturbance. However, the areas with highest plant diversity are not *per se* the areas with largest soil biodiversity [8]. Recent work, enabled by molecular techniques to study soil biodiversity and joint efforts between scientists, resulted in datasets that start to reveal the global distribution and potential drivers of soil biodiversity. Below, we highlight a number of those studies.

Globally, earthworm species richness and abundance is linked to climate, being largest in temperate regions, yet also soil characteristics play an important role ([11] with erratum in 2020). Earthworms are most abundant in grassland and temperate deciduous forest soils, whereas in acid wet soils as in tundra and boreal forests enchytraeids thrive [12]. Soil nematodes are most abundant in sub-Arctic regions, followed by temperate and tropical regions [13].

The global distribution of nematodes appears to relate more strongly to soil conditions, such as organic matter content, than to climatic conditions. The distribution of nematode functional groups (bacterivores, fungivores, omnivores, carnivores and plant-feeders) remained consistent across the globe, indicating general patterns in soil food-web composition at the functional level. Bastida *et al*. [14] showed that the most diverse soil invertebrate groups across the globe are nematodes, arachnids and rotifers; their diversity primarily being associated with (lack of) aridity and plant diversity and productivity. Nematodes and rotifers live in water films which can explain their sensitivity to aridity. Arachnids, in the study primarily soil mites, live in the non-water-filled soil voids. Their sensitivity to aridity may be owing to reduced food availability, although they require a minimum soil humidity level to survive and reproduce.

With respect to the global distribution of topsoil bacteria Delgado-Baquerizo *et al*. [15] found that a few dominant taxa (representing 2% of the diversity) make up nearly half of all the bacterial communities. These generalist taxa can be subdivided in clusters of co-occurring bacterial taxa according to different habitat preferences. In the global-scale study of Bahram *et al*. [16], topsoil bacterial and fungal diversity, community structure and functional potential (based on functional genes for substrate utilization) were investigated. Bacteria and fungi were found to relate differently to global environmental gradients. Bacterial taxonomic and functional diversity peaked in temperate habitats. By contrast, fungal taxonomic diversity declined and biomass increased from the equator towards the poles and fungal functional diversity was lowest in temperate regions. Bacterial taxonomic diversity and abundance related mostly positive to soil pH, soil nutrient levels (low soil carbon (C) : nitrogen (N)) and mean annual precipitation (MAP). Fungal functional composition and biomass were higher at higher soil C : N, suggesting globally higher substrate specialism of fungi as compared to bacteria. Communities of topsoil bacteria and fungi result from environmental filtering and from competition between bacteria and fungi, as evidenced by the prevalence of antibiotic-resistance genes. However, at a global-scale, fungi and bacteria show different niches in terms of soil pH, MAP and soil C : N ratio. Note that the global-scale studies are at relatively coarse taxonomic scale, and in bulk soil plant symbionts are generally less abundant than saprotrophic bacteria and fungi. Abundances of plant species-specific pathogens and symbiotic mutualists are primarily determined by plant host abundance and dispersal mode [17–20]. For the global distribution of mycorrhizal fungi, there is a clear distinction between arbuscular mycorrhizal fungi (AMF) and ectomycorrhizal fungi (EcM). EcM are taxonomically more diverse yet colonize fewer plant species than AMF which are taxonomically less diverse but colonize most vascular plant species [21]. Furthermore, EcM fungi are most prevalent in roots of plants growing on acidic soils and in areas with fairly constant precipitation levels [19,22]. By contrast, AMF proliferate most in plants grown in continental climates and mild summers, on soils with relatively high N content (low soil C : N ratio). In terms of taxonomic diversity AMF comprise taxa with a nearly global distribution, whereas other taxa are confined to specific habitats and plant species [23].

Overall, abundances and composition of soil microbial and faunal communities are related to climatic and soil conditions, similarly to the vegetation with which they interactively create and maintain habitats. However, the pattern of increased taxonomic richness from the poles towards the equator as observed for plants does not hold for soil biota. The co-occurrence of specific vegetation types and soil organisms varies with the respective lifestyles of the soil biota. This ranges from widely distributed, easily dispersing generalist decomposers to more specialistic root symbionts or less mobile and climate sensitive soil biota with more constraint geographical distribution [12,24]. At a local scale similar soil parameters as in the global surveys appear to be main drivers of the composition of microscopic soil communities, notably soil pH [25] and soil texture [26]. Locally, root associated microorganisms are primarily recruited from the bulk soil, hence soil management that shapes soil microbiomes is of key importance to the development of plant microbiomes [27].

## 3. Role of soil biota in soil formation and habitat creation

**Figure 1.**
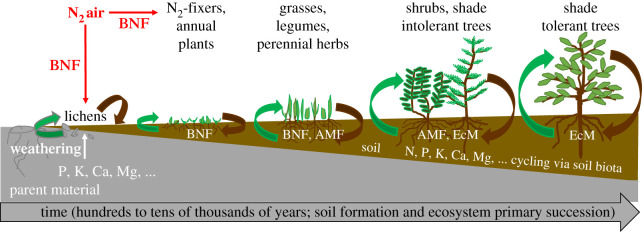
Primary succession and soil formation are interdependent through soil–soil biota–plant interactions and feedbacks, with complementarity in the carbon and nutrient cycling. Left to right: soils form over time becoming deeper and richer in organic matter and nutrients. Symbiotic interactions play an important role in this, notably biological nitrogen fixation (BNF), arbuscular mycorrhizal fungi (AMF) and ectomycorrhizal fungi (EcM). Green arrows indicate feedback to autotrophs from the soil, brown arrows indicate feedbacks from autotrophs to the soil in terms of flows of nutrients and carbon as litter is returned.

**Figure 2.**
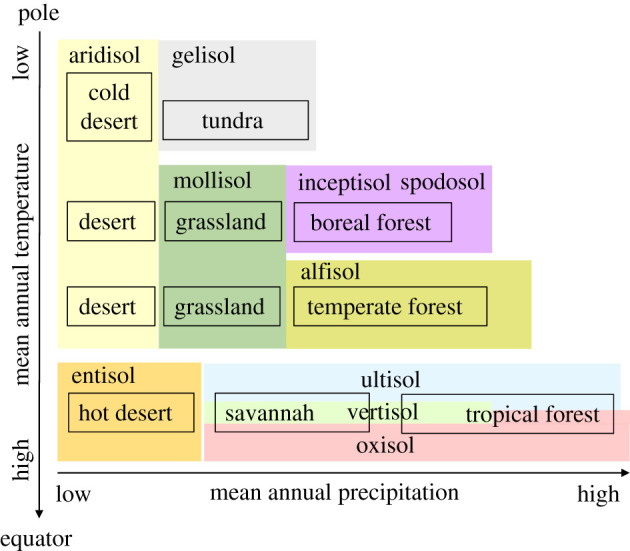
Main biomes and their main soil types (orders) across the globe from the equator to the poles, as influenced by climatic factors of mean annual precipitation and temperature. (Online version in colour.)

Soil formation from the parent rock into deep, fertile, carbon-rich and biodiverse soil is generally a slow process and requires intimate feedback interactions between soil life, plants and soil physical and chemical properties [4,28,29] (figure 1). Formation of new soil starts with rock weathering by the physical impacts of water and changing temperatures and through biogeochemical weathering by lichens. Lichens bring C and nutrients into the soil as mutualistic symbiosis between cyanobacteria and fungi. The cyanobacteria fix C via photosynthesis and N by biological N fixation (BNF) and the fungi extract mineral nutrients from the rock. In soil N is an exceptional nutrient, its primarily source is the air, not rocks. When the bedrock origin is sedimentary instead of igneous it can contain significant amounts of N [30]. In young soils, N is the main plant growth limiting nutrient, while heterotrophic soil life is constrained by C availability. Over time a rootable soil layer is formed and N_2_-fixing plants thrive as their symbiotic root bacteria fix N and other nutrients are available in the soil [28]. Short lived, easy dispersing plants also appear, taking advantage of the soil nutrients and the absence of light competition. When soils are more developed longer living plants that grow taller and deeper can establish. This increases light competition, but also root proliferation, litter input, plant defences, and diverse root symbiont strategies for nutrient acquisition from soil [24,29]. Furthermore, symbiotic root fungi can speed up mineral weathering, thereby enhancing nutrient access [31,32]. Using long-term chronosequences, Lambers *et al*. [33] showed general patters in succession of species of plant symbioses with AMF, followed by EcM and then ericoid mycorrhiza, as soils develop from being poor in N and rich in phosphorus, to rich in N and poor in phosphorus. In very old, nutrient depleted soils atmospheric dust deposits become the major source of many minerals for plants [28]. These studies demonstrate the importance of the nutrient sources, the different types of root symbionts for plant access to these nutrients and the essential role of litter decomposition and mineralization by the soil food-web during succession (figure 1). Also, soil engineers namely plant roots, fungi, earthworms and termites that help to physically build soil structure and prevent erosion are cornerstones of soil habitat formation and maintenance. The type of prevalent soil invertebrate ecosystem engineers and root symbiotic fungi depend on the biome and soil type owing to different tolerances for soil pH, drought, temperature and (host) plant presence [12,22]. The successional trajectory as described above is constraint by climatic conditions, for example forests require a minimum level of annual precipitation (figure 2). Moreover, successional development over time is not *per se* linear or unidirectional. For example, when an aridity threshold is reached, sudden losses in soil functioning, soil biota and plant cover occur [34].

Soils develop over time and this development depends on climate and vegetation type. At the same time different biomes from the equator to the poles are not only associated with different climate, but also with different soil types (figure 2; modified from [22,12]). This schematic soil representation is at coarse taxonomic level and is based on the United States Department of Agriculture soil taxonomy [35]. We recognize that within each soil order many soil types can be distinguished, but that level of detail is beyond the scope of this manuscript. The role of different soil biota in habitat creation differs between soil types and biomes because, as described in §2, the distribution of different soil biota varies across biomes. This is especially the case for invertebrate soil fauna as they are more dispersal limited than soil bacteria and fungi, and more drought and temperature sensitive. Soil invertebrate ecosystem engineers contribute a lot to habitat creation by organic matter relocation and mixing with mineral soil, deepening the soil, creating physical structures and are food for predators [5,12]. Earthworms are well known for these functions in temperate grassland (mollisol) and forest (alfisol) soils. In the cold, wet and acidic tundra (gelisol) and boreal forest (inceptisol and spodosol) soil enchytraeids replace earthworms. In soil of arid areas (aridisol and entisol), provided there are high enough temperatures, termites are the prime soil engineers. Termites are active in savannah and tropical forest (mostly in ultisol, oxisol) soils and in tropical forests complement earthworms and ants. Ants are also active as soil engineers and mediators of seed dispersal in temperate grassland and temperate and boreal forest soils. Note that each group of soil engineers comprises numerous species each with their own habitat preference and feeding strategy, and sensitivity to environmental change [12].

Soil properties and climate are inter-related so disentangling their impact on soil communities is challenging. To this end, Laliberté *et al*. [36] studied soil food webs along four soil chronosequences from soil build-up to retrogression, each spanning a strong regional climate gradient. The study showed that changes in belowground communities were owing to changes in soil fertility rather than climate. Biomass of soil fungi and bacteria peaked at intermediate stages of the chronosequences as did soil organic matter level. The change in microbial biomass also showed bottom-up effects on the higher trophic levels in the soil. Along the same line, Delgado-Baquerizo *et al*. [37] studied sixteen chronosequences across the globe. This meta-analysis showed that parent material type, climate, vegetation and topography have an over-ruling impact on ecosystem structure, and functioning and soil age plays a minor role. These studies show the major impact of environmental context on the development of living soils and ecosystem structure and functioning. However, these studies focused on the top 10 cm soil layer. Also in the soil below this top layer soil biota and roots play critical roles in habitat formation and maintenance, stressing the need to dig deeper to reveal the full role of roots and other soil biota [38–40].

## 4. Human use of soils: habitat destruction

Since the onset of agriculture and development to sedentary life (±11 000 years ago) humans depend on soil and have started to alter their environment. Fertile alluvial soils along riverbanks were prime locations for successful agriculture and population expansion. Replenishment of soil nutrients probably relied on crop residues, BNF and animal manure, and on sediment deposited after flooding of soils along riverbanks. Especially in areas with poor soils manure use was essential for agricultural expansion, as shown for the Loess Plateau in China [41]. Also in the Mediterranean Basin early farmers managed their land, yet this could not prevent a decline in soil fertility [42]. Throughout human agricultural history there are numerous examples of civilization collapses owing to misuse of their soil resources [43]. Current societies also face a severe decline in soil resources, as outlined in the assessment report on land degradation and restoration of the Intergovernmental Science-Policy Platform on Biodiversity and Ecosystem Services (IPBES) [44]. The issue is not just nutrient imbalances, but the cascading impacts of changing the natural habitat by vegetation clearing resulting in loss of soil stability, fertility, water retention and soil life, as the natural plant–soil feedbacks are broken.

The impact of transforming natural habitats for agricultural use depends on the habitat, soil type, topography, climate and scale. For example, mollisols and alfisols are inherently more fertile than ultisols and oxisols. The large aboveground standing biomass of tropical rainforests may suggest that they grow on rich soils. Yet the opposite is true, their soil is old and weathered and only through the intricate above- belowground interactions that co-evolved over long periods of time can the aboveground diversity and productivity be maintained. Conversion of these habitats is virtually irreversible and leads to major biodiversity loss and loss of soil C that accumulated over thousands of years [45], it also yields only marginal land for agriculture. Even though many humans now live in cities soils are still the basis of our food system. With expanding populations, economic development and global trade, our impact on soils and other natural resources is no longer just in our backyard, but more and more cross continental as consumption increases [46]. This spatial disconnection between consumers of resources (institutions, companies, individuals) and the impacts of the resource extraction on local habitat and soil degradation, is one of the main reasons why land degradation is ongoing [47].

Humans have been very inventive in modifying their habitat, not only physically but also chemically. The notion that crop growth is primarily limited by N (in soils that are not phosphorus depleted) and the invention of the Haber–Bosch process to convert atmospheric N_2_ into ammonia enabled large increases in food production. However, owing to the rate of mineral N applications to soils globally and the mobility of several N-forms, many unwanted side-effects appeared on biodiversity, soil and water quality and N-emissions [48,49]. As a consequence, the natural soil–plant interactions and feedbacks are disrupted and the new conditions promote just a few common species at the expense of plant diversity and their associated organisms above- and belowground [50]. As shown by the Intergovernmental Panel on Climate Change (IPCC) in the Special Report on Climate change, desertification, land degradation, sustainable land management, food security and greenhouse gas fluxes in terrestrial ecosystems [51]; these global issues are interlinked, and soils are key to the solution as the basis for terrestrial biodiversity, food production and sink of greenhouse gases.

## 5. Soil and habitat conservation and regeneration

As already predicted by Sala *et al*. [52] habitat destruction owing to land-use change for agriculture and lodging is now the main cause of terrestrial biodiversity loss [53], including loss of soil biodiversity, ecosystem services and regeneration potential [44,54]. Soil conservation and regeneration is urgent for biodiversity and human well-being [44]. Historical large-scale examples show soil regeneration is possible, provided there is adequate policy and governance. For example, to combat the American Dust Bowl the United States (US) government passed the Soil Conservation Act in 1935 (amended in 1936). Herewith land owners and farmers received financial support from the government for applying practices to combat soil erosion, such as planting trees, grasses and legumes. Next to soil conservation and regeneration the aim was safe-guarding farmers income and availability of food for all US citizens. More recently, in 1994 the restoration of the Loess Plateau in China started. The Loess Plateau Watershed Rehabilitation Project aimed at ecological as well as economic restoration and was enabled with support of The World Bank, in partnership with the Chinese government. Restoration success required good policies, governance and participation of the local people to include their knowledge, and to change behaviour and avoid tragedy of the commons in the land use. The project benefitted from biological and technological innovations with targeted replanting schemes to promote soil stabilization, and terracing of landscape parts for low erosion risk cultivation and higher crop yields. Restoration of the Loess Plateau is still ongoing, with special attention for local plant species and climate change projections [55,56]. Also in Europe there are positive signals; in the recently proposed European Green Deal, soils are identified as key element for achieving the ambitious European target of a climate neutral European Union by 2050, while sustaining the role of soils as a large biodiversity pool [57].

Soil and habitat conservation and regeneration start with databased geo-referenced knowledge of the status of soils and the pressures exerted on them so that leverage points can be pinpointed. The 2015 report ‘Status of the World's Soil Resources’ from the United Nations Food and Agriculture Organization Global Soil Partnership (UN-FAO-GSP) showed that globally one-third of the soils are degraded, primarily owing to erosion, salinization, chemical pollution and urbanization [58]. Consequently, soil biodiversity lost habitat and is under threat, yet data on soil biota are sparse and large areas of the globe are unexplored [9,59]. Also the reports of IPBES [44] and IPCC [51] provide a reference for the current status of biodiversity, land degradation, climate change and their interlinkages. In economic terms, the general picture is that costs of restoration of natural habitats are often larger than those of conservation [44]. Moreover, climate change risks make soils an even more valuable non-renewable resource and should as such be included into economic projections of world economic development [60]. The need for responsible production and consumption to safeguard and restore our natural habitats is recognized in international global agreements such as the Sustainable Development Goals (SDGs), launched in 2015 by the UN. To make change happen clear targets need to be set, along with quantifiable indicators that enable monitoring of progress and to evaluate the impact of interventions. For example, SDG 15.3.1 (proportion of land that is degraded over total land area) aims to combat desertification and land degradation and to promote soil and habitat conservation and restoration. Thereto three main indicators were developed: land cover and land cover change; land productivity; and above- and belowground C stocks. To quantify these indicators at high spatial and temporal resolution remote sensing technology is a powerful tool [61–63]. However, remotely sensed indices require integration with solid ecological knowledge to be effective and to avoid undesirable side-effects [64]. The most appropriate sensing methods also depend on the required resolution and specific properties that are aimed at [65].

Natural and agricultural habitats differ in many respects and we need both for sustainable development. Coexistence of different habitat types requires adequate landscape management to avoid imposing stress and to attain multifunctionality. Natural habitats remain essential for biodiversity conservation as they comprise co-evolved and interlinked above- and belowground biodiversity and elemental cycles. These systems are also essential for soil conservation, C uptake and water purification and storage. The size of natural habitat fragments is of prime importance for conservation success. Small fragments suffer ecosystem decay, thereby host disproportionally less biodiversity than larger habitat fragments [66] and have reduced ability for C uptake [67]. Agricultural systems are designed to produce food, feed and fibre, which creates physical disturbance and extraction of energy, nutrients and water. However, the level of disturbance and thereby the impact on soil habitat quality is strongly dependent on the type of land management. For example, grasslands host more biodiversity and promote soil organic matter build-up compared to arable fields, whereas minimal/no-till and growth of cover crops promote soil conservation in arable fields [9,54,59]. Agro-ecosystems can be biodiverse habitats through diversification of the plants grown within and surrounding the fields. Moreover, within landscapes well-designed mosaics of higher and lower land-use intensity and natural corridors to connect conservation areas can provide the required multifunctionality in terms of sustaining food production and providing diverse habitats [68]. Additionally, diversified agro-ecosystems can use N resources more efficiently and offset greenhouse gas emissions [69].

**Figure 3.**
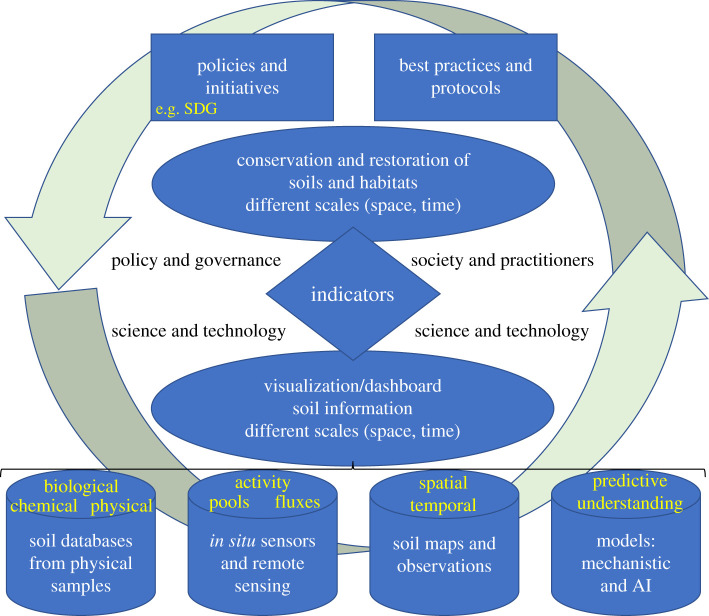
Schematic of the governance cycle, relevant stakeholders and available data sources to support the conservation and restoration of soils and habitats at varying spatial and temporal scales. (Online version in colour.)

Regeneration of degraded soil starts with identifying and lifting the pressure(s) that lock(s) the soil in a degraded state. This may for example be overgrazing, chemical pollution or erosion which can be lifted by excluding grazers, removing the pollutant, or by breaking wind and water force and fixing soil by growing perennial plants. Subsequently, beneficial plant–soil feedbacks can be restored to move from a soil degradation to a soil regeneration trajectory, to promote diversity, resilience, resource use efficiency and productivity of natural and agricultural systems [70]. This requires the presence of plants and associated root and soil organisms with the required traits to grow under the prevailing conditions, and traits that enhance soil physical, chemical and biological properties. These organisms may still be present in the soil or need to be (re-)introduced, for example, via soil inoculation with soil from a matching donor site [71]. The regeneration trajectory of the soil microbiome and associated plant community is yet hard to predict, as we are just starting to discover the couplings between plant and soil biota taxonomic composition, the processes they generate and their dependence on environmental conditions [27]. These studies are challenging because soil communities can vary strongly at small spatial scales and are temporally variable [7,10,72]. Recent efforts in unifying methodologies and databases of (soil) microbial composition and environmental parameters, along with microbial metabolic trait databases, are promising to gain deeper understanding of the hitherto hidden diversity (e.g. [73,74]). New tools that enable *in situ* observations at scale at high resolution in space and time, such as remote sensing via satellites or drones, offer great potential to support habitat restoration and regenerative soil use. Soils are opaque, yet remote and proximal sensing of bare topsoil and of plants responding to soil variation enables the characterization of soil variation for precision agriculture, limiting negative side- effects of fertilizer use and pest control [75]. Moreover, these technologies can help to better understand and quantify plant–soil feedback interactions in the field and to integrate beneficial ecological interactions in land management from local to regional scale [69,76,77]. Also for monitoring biodiversity, sensing technology is an asset especially when used in combination with *in situ* activity sensors and DNA barcoding [78,79]. Sensing technology warrants solid parametrization with *in situ* collected data which are labour and data intensive, yet these investments pay off as precision increases with more solid datasets. This development is supported by efforts to streamline methods and datasets via global scientific community initiatives such as the Group on Earth Observations Biodiversity Observation Network (GEOBON; with Soil BON as one of its thematic networks in collaboration with the Global Soil Biodiversity Initiative) and the Food and Agriculture Organization's Global Soil Laboratory Network (GLOSOLAN). To adequately collect and interpret these datasets in the context of the multiple facets of biodiversity and soils, ecological and soil knowledge remains indispensable [79]. The best indices for soil health or soil quality assessments also depend on the aim of the stakeholders and co-development is advised [80]. For effective conservation and restoration of soils and the habitats they support, scientific knowledge and technology are indispensable, yet not enough (figure 3). With a shared goal and associated indicators for soil and habitat conservation and restoration scientists, policy makers, society and private partners can join forces acting complementary, provided the processes/sub-systems are in tune. To bend the curve we need a systems change across economic, social and political systems such that sustainable land management becomes the norm and replaces destructive practices [47,50,81]. Citizens, policy makers and scientists all can contribute each in their own spheres of influence and can enforce each other [47]. The value of soils, biodiversity and habitats goes beyond economic value, we have an ethical duty to preserve these for future generations, also as source of wonder and inspiration. Especially now many people live in cities we need to ensure the connections with the natural world remain. For example, via public city parks, which at the same time combat urban heat islands, improve air quality and water infiltration and provide a habitat for above- and belowground biodiversity [25]. Also school gardens can help raise awareness and support youngsters (and their parents) to learn about soils, biodiversity, nutrient (re)cycling and pollution. Incentives such as ‘Nature based solutions' [82], the SDGs and UN Decade on Ecosystem Restoration can play a leading role in concerted action and aligned vision for soil and habitat restoration for the benefit of nature and humanity, together with participatory approaches that include knowledge, values and needs of all stakeholders [83]. Scientists' primary role remains proving solid and objective data, ecological insights and tools to capture the status of the world and project scenarios, yet also play an important role in raising awareness and urge to action among policy makers, business and the general public.

## 6. Conclusion

As already recognized in the eighteenth century by James Hutton, present soils are key to the past [84]. Soils harbour a rich history and diversity and are at the basis of terrestrial life on the Earth. Soils are dynamic in space and time and the formation of soils that support rich habitats and food production took hundreds to tens of thousands of years to form. However, in the last half century habitat and soil degradation primarily owing to land-use change caused soil losses at much faster rates than new soil was formed. This trajectory is compromising natural habitats, food security and quality of life. The curve can be bent and habitats and soils can be conserved and restored, yet this requires concerted action and a systems change. Investing in soils, closing nutrient cycles and sustainable soil management can generate multiple win–wins when we consider all the costs and benefits of our current and alternative modes of operation. The way we start managing our soils now is key to the future.

## Data accessibility

This manuscript does not contain new primary data.
